# Multidisciplinary Management of Complicated Crown-Root Fracture: A Case Report

**Published:** 2018-05

**Authors:** Zahra Enshaei, Maede Ghasemi

**Affiliations:** 1 Assistant Professor, Dental Research Center, Department of Pediatric Dentistry, School of Dentistry, Isfahan University of Medical Sciences, Isfahan, Iran; 2 Assistant Professor, Dental Materials Research Center, Department of Operative Dentistry, School of Dentistry, Isfahan University of Medical Sciences, Isfahan, Iran

**Keywords:** Tooth Injuries, Tooth Fractures, Orthodontic Extrusion

## Abstract

The management of traumatic dental injuries as well as crown-root fractures is always challenging in everyday general dental practice. A number of treatment modalities are available for crown-root fractures, depending on the position, extent and severity of the fracture. The aim of this case report was to describe a clinical case of rehabilitation of a complicated crown-root fracture of the maxillary left central incisor, successfully treated by a multidisciplinary approach including orthodontic extrusion. The final result was esthetically pleasant and periodontally sound in the follow ups.

## INTRODUCTION

Dental injuries predominantly occur during the first two decades of life with the majority of these injuries affecting the maxillary incisors [1,2]. Crown-root fracture is a type of fracture that involves the enamel, dentin and cementum. Crown-root fractures extending apically towards both the gingival margin and the alveolar crest pose a great challenge [3,4]. Herein, we report a clinical case of rehabilitation of a complicated crown-root fracture, using a multidisciplinary approach.

## CASE PRESENTATION

A 10-year-and-8-month-old male patient who presented emergently with a history of falling while playing soccer in a playground was admitted to the Department of Pediatric Dentistry, School of Dentistry, Isfahan University of Medical Sciences. His medical history was unremarkable.

**Fig. 1:**
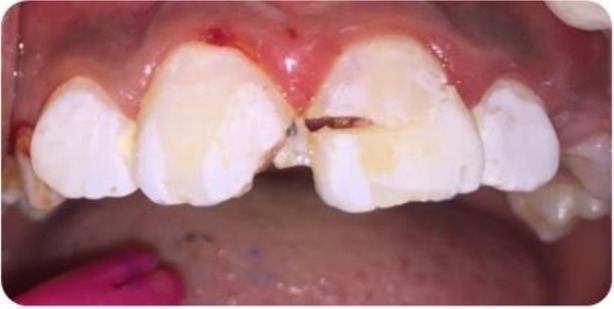
Intraoral buccal view of the traumatized maxillary central incisors with complicated(left) and uncomplicated(right) crown fracture.

**Fig. 2:**
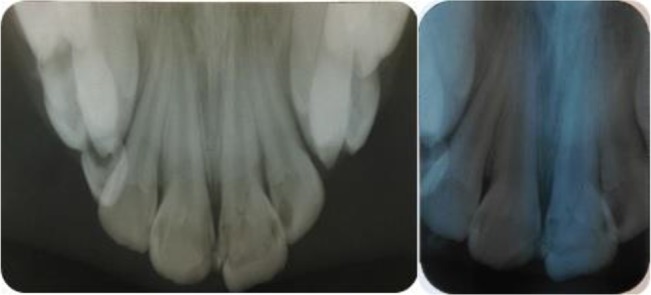
Preoperative intraoral occlusal(left) and periapical(right) radiographs demonstrating oblique complicated crown-root fracture affecting the left central incisor

**Fig. 3:**
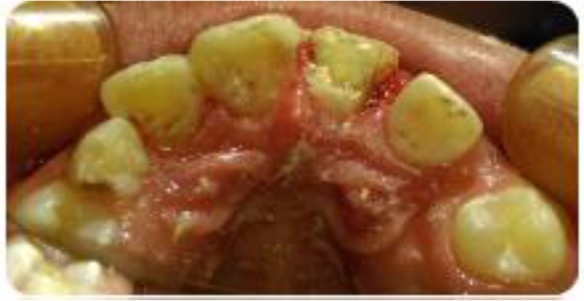
Intraoral occlusal view after separating fractured segment

**Fig. 4:**
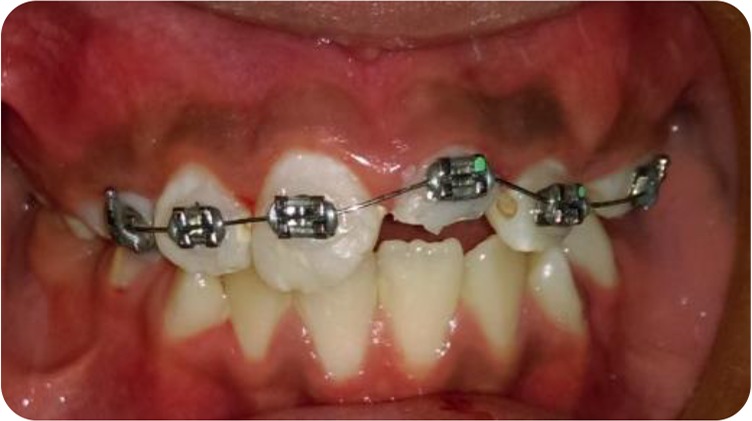
Intraoral view of the orthodontic extrusion with brackets

**Fig. 5:**
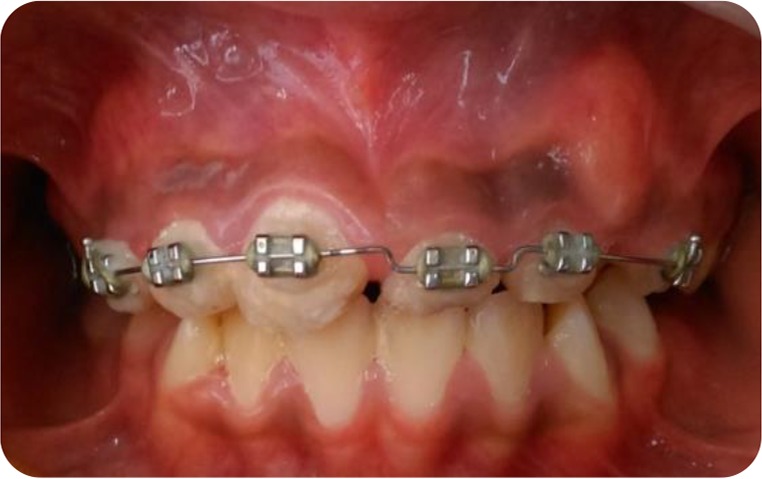
Intraoral view at the end of the orthodontic extrusion

Intraoral examination revealed a complicated crown-root fracture and an uncomplicated crown fracture of the maxillary left and right central incisors, respectively (Fig. 1). The teeth were slightly tender on percussion with no associated mobility and had normal response to vitality tests. Radiographic examination revealed an oblique fracture line in the maxillary left central incisor, ending at the cervical third of the root; the root was fully developed (Fig. 2), and had no periapical pathosis or displacement. After obtaining an informed consent, an emergency treatment was undertaken to stabilize the coronal fragment by splinting it to the adjacent teeth using acid-etch/resin and sealing the fracture line with flowable composite resin (Grandio Flow; Voco, Cuxhaven, Germany). At the second visit, pulpectomy with a working length of 27 mm was performed, and calcium hydroxide (Ultracal XS; Ultradent, South Jordan, UT) paste was placed as an intracanal medicament, with the access cavity being sealed until definite treatment. During the third visit, the root canal was filled, followed by temporary restoration of the tooth with glass-ionomer restorative material (Fuji IX; GC Corporation, Tokyo, Japan). After root canal therapy of the maxillary left permanent central incisor, the fractured part was separated to assess the fracture line, which revealed that it was extended subgingivally for about 2.5 mm distally (Fig. 3). To expose the fracture margins supragingivally, it was decided to extrude the fractured tooth via an orthodontic procedure. After oral prophylaxis, brackets were bonded to the upper teeth on a straight line from the primary right canine tooth to the left first permanent premolar tooth except for the tooth which had to be extruded. Subsequently, 0.014-inch Ni-Ti flexible wire was used for 2 weeks (Fig. 4). Leveling of the maxillary incisors of the patient continued by reactivation with 0.016-inch SS wire and the result of short-term fixed orthodontic treatment (4 mm extrusion in 5 weeks) is shown in Figure 5.

**Fig. 6:**
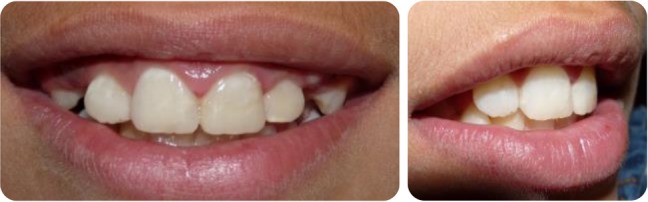
Final result of resin composite build up

**Fig. 7:**
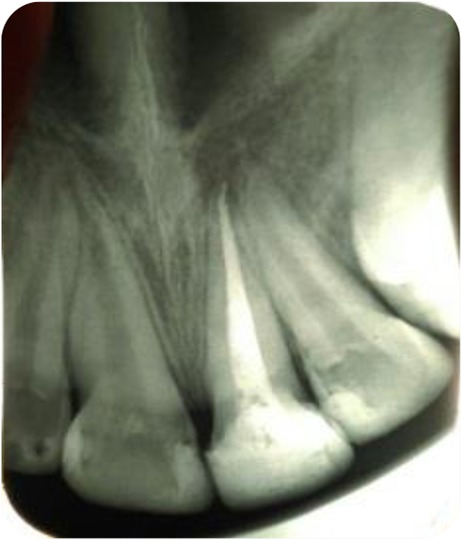
Periapical radiograph at 12-month follow-up

To avoid relapse, a circumferential supracrestal fiberotomy, extending below the level of marginal bone, was performed prior to the retention period. After the retention period which lasted for 4 weeks, debonding was performed. The root canal was prepared for intracanal post placement by post drills; then, dual-cure self-adhesive resin cement (Clearfil™ SA Cement; Kuraray Noritake Dental, Tokyo, Japan) was placed in canal by a Lentulo drill. At that time, a fiber post (RelyX™ Fiber Post; 3M ESPE, St. Paul, MN, USA) was placed and excess cement was removed before light-curing. Then, the tooth was restored completely with composite resin (Fig. 6) with both enamel- (Amaris® Enamel shades Translucent TN; Voco, Cuxhaven, Germany) and dentin-like materials (Amaris® Base shades Opaque O1; Voco, Cuxhaven, Germany) using the incremental technique. A custom-made mouth guard was fabricated for patient to prevent further trauma. In routine follow-up appointments after 3 and 12 months, clinical and radiographic examinations showed healthy tissues and teeth, and no evidence of apical periodontitis was seen (Fig. 7).

## DISCUSSION

Early loss of an incisor in a child might bring about esthetic and psychological problems; in addition, it might result in the development of malocclusion, with negative effects on the alveolar bone [5]. A treatment plan involving different dental specialties (i.e. pedodontics, endodontics, oral surgery, orthodontics and operative dentistry) must be designed for optimal treatment of the affected teeth [6]. Implementing the restorative principles together with proper management of periodontal tissues can ensure long-term survival of the injured tooth [7]. A number of treatment modalities are available for crown-root fractures, depending on the position, extent and severity of fracture [8], which include a crown lengthening procedure so that the crown margins would be exposed, orthodontic extrusion, surgical repositioning (intentional replantation), restorative management of subgingival margins, decoronation (root submergence) and extraction [9]. During the treatment planning, the risks and benefits of each treatment alternative should be carefully assessed. Crown lengthening is fraught with compromise particularly in the anterior segment where esthetics is a concern [10].

The biologic width should not be impinged upon while placing the restorative margins because it is a potential source for failure of restoration as it might give rise to irreversible damage, involving gingival inflammation, resorption of the alveolar crest and recession [11]. Regarding the extrusion methods of fractured tooth for exposing the fracture margins supragingivally, orthodontic extrusion is the most favorable technique biologically, followed by surgical repositioning. However, surgical repositioning is always associated with a risk of ankylosis [12]. On the other hand, orthodontic extrusion requires several visits and excellent patient cooperation while surgical extrusion is a procedure which is simpler and less time-consuming [13]. During rapid orthodontic extrusion, the periodontal fibers are stretched and readjusted; therefore, marked bone remodeling is avoided during rapid movement. Consequently, coronal restoration is facilitated due to a lack of need for reshaping of the bone [4].

Patient age, fracture size, occlusion, parafunctional habits, esthetic requirements, financial status and the dentist’s dexterity should be considered in treatment of a fractured front tooth [4]. Full coverage of the tooth should be avoided in young adolescents or during the early adult dentition period since young anterior teeth may not be fully erupted, and the gingiva may not respond favorably to the placement of a crown margin below the free gingival margin [4,14]. The initial management of a fractured incisor tooth in most cases consists of a crown build-up with the use of composite resin.

## COUCLUSION

Despite the difficulties and the complexities, the majority of teeth with crown-root fractures can be saved with combined efforts of endodontists, orthodontists, periodontists and prosthodontists. This case report demonstrated the importance of a multidisciplinary approach to meet the esthetic and functional requirements for traumatized maxillary central incisors.

## References

[1-] Andersson L Epidemiology of traumatic dental injuries. J Endod. 2013 3;39(3 Suppl):S2–5. 23439040 10.1016/j.joen.2012.11.021

[2-] Ravindranath S Andiesta NS Hasan ZA Chong JA Pau A Patterns of dental trauma in children presenting in hospital based dental clinics: A review. Dental Health: Current Research. 2016 7;2016.

[3-] Diangelis AJ Andreasen JO Ebeleseder KA Kenny DJ Trope M Sigurdsson A Guidelines for the management of traumatic dental injuries: 1. Fractures and luxations of permanent teeth. Pediatr Dent. 2017 9;39(6):401–11. 29179382 10.1111/j.1600-9657.2011.01103.x

[4-] Andreasen JO Andreasen FM Andersson L Textbook and color atlas of traumatic injuries to the teeth. Oxford, UK, Ames, Iowa, Blackwell Munksgaard, 2007:684–90.

[5-] Yuan LT Duan DM Tan L Wang XJ Wu LA Treatment for a complicated crown-root fracture with intentional replantation: a case report with a 3.5-year follow up. Dent Traumatol. 2013 12;29(6):474–8. 22453056 10.1111/j.1600-9657.2012.01130.x

[6-] Acharya N Samant PS Gautam V Singh O Halwai H Multidisciplinary approach in the rehabilitation of complicated crown-root fracture: A Case report. Orthod J Nepal. 2015 11;4(2):51–5.

[7-] Mese M Akcay M Yasa B Akcay H Multidisciplinary management of complicated crown-root fracture of an anterior tooth undergoing apexification. Case Rep Dent. 2015;2015:521013. 26146573 10.1155/2015/521013 PMC4471325

[8-] Heithersay G Moule A Anterior subgingival fractures: a review of treatment alternatives. Aust Dent J. 1982 12;27(6):368–76. 6963152 10.1111/j.1834-7819.1982.tb02467.x

[9-] Aggarwal V Logani A Shah N Complicated crown fractures–management and treatment options. Int Endod J. 2009 8;42(8):740–53. 19548932 10.1111/j.1365-2591.2009.01588.x

[10-] Bajaj P Chordiya R Rudagi K Patil N Multidisciplinary approach to the management of complicated crown-root fracture: a case report. J Int Oral Health 2015 4;7(4):88–91. PMC4409806 25954080

[11-] Padbury A Eber R Wang HL Interactions between the gingiva and the margin of restorations. J Clin Periodontol. 2003 5;30(5):379–85. 12716328 10.1034/j.1600-051x.2003.01277.x

[12-] Bach N Baylard JF Voyer R Orthodontic extrusion: periodontal considerations and applications. J Can Dent Assoc. 2004 12;70(11):775–80. 15588553

[13-] Roeters J Bressers JP The combination of a surgical and adhesive restorative approach to treat a deep crown-root fracture: A case report. Quintessence Int. 2002 3;33(3): 174–9. 11921763

[14-] Padbury A Eber R Wang HL Interactions between the gingiva and the margin of restorations. J Clin Periodontol. 2003 5;30(5):379–85. 12716328 10.1034/j.1600-051x.2003.01277.x

